# Mechanisms of socioeconomic differences in COVID-19 screening and hospitalizations

**DOI:** 10.1371/journal.pone.0255343

**Published:** 2021-08-05

**Authors:** Jarrod E. Dalton, Douglas D. Gunzler, Vardhmaan Jain, Adam T. Perzynski, Neal V. Dawson, Douglas Einstadter, Yasir Tarabichi, Peter B. Imrey, Michael Lewis, Michael W. Kattan, James Yao, Glen Taksler, Kristen A. Berg, Nikolas I. Krieger, David Kaelber, Lara Jehi, Ankur Kalra

**Affiliations:** 1 Cleveland Clinic Lerner College of Medicine at Case Western Reserve University, Cleveland, Ohio; 2 Case Western Reserve University at MetroHealth Medical Center, Cleveland, Ohio; Sciensano, BELGIUM

## Abstract

**Background:**

Social and ecological differences in early SARS-CoV-2 pandemic screening and outcomes have been documented, but the means by which these differences have arisen are not well understood.

**Objective:**

To characterize socioeconomic and chronic disease-related mechanisms underlying these differences.

**Design:**

Observational cohort study.

**Setting:**

Outpatient and emergency care.

**Patients:**

12900 Cleveland Clinic Health System patients referred for SARS-CoV-2 testing between March 17 and April 15, 2020.

**Interventions:**

Nasopharyngeal PCR test for SARS-CoV-2 infection.

**Measurements:**

Test location (emergency department, ED, vs. outpatient care), COVID-19 symptoms, test positivity and hospitalization among positive cases.

**Results:**

We identified six classes of symptoms, ranging in test positivity from 3.4% to 23%. Non-Hispanic Black race/ethnicity was disproportionately represented in the group with highest positivity rates. Non-Hispanic Black patients ranged from 1.81 [95% confidence interval: 0.91–3.59] times (at age 20) to 2.37 [1.54–3.65] times (at age 80) more likely to test positive for the SARS-CoV-2 virus than non-Hispanic White patients, while test positivity was not significantly different across the neighborhood income spectrum. Testing in the emergency department (OR: 5.4 [3.9, 7.5]) and cardiovascular disease (OR: 2.5 [1.7, 3.8]) were related to increased risk of hospitalization among the 1247 patients who tested positive.

**Limitations:**

Constraints on availability of test kits forced providers to selectively test for SARS-Cov-2.

**Conclusion:**

Non-Hispanic Black patients and patients from low-income neighborhoods tended toward more severe and prolonged symptom profiles and increased comorbidity burden. These factors were associated with higher rates of testing in the ED. Non-Hispanic Black patients also had higher test positivity rates.

## Introduction

Differences in SARS-CoV-2 infection rates, COVID-19 hospitalizations, and COVID-19 deaths relating to race, ethnicity and geography have been documented worldwide [1–11]. These alarming disparities were noted early in the pandemic in the UK and USA [12,13]. However, the underlying relationships that may operate to produce these differences are less understood.

In the USA specifically, Black or African American persons are substantially more likely to contract, be hospitalized, and die of COVID-19 than their White counterparts [3]. Geographically, COVID-19 has disproportionately impacted regions with more racial and ethnic minority representation [3,5,6,10,11,14]. Racial differences in risk of death from COVID-19 are likely complex and multifactorial [15–20]. These differences do not appear to be fully accounted for by variations in area-based socioeconomic position [6], or medical comorbidities [2,4].

The goal of this study was to further understand the social and biological processes that underlie these ecological differences. Specifically, we identified and analyzed potential mechanisms of social and geographic differences in early (March-April 2020) SARS-CoV-2 testing access, test positivity, and COVID-19 hospitalization rates, among a regional cohort of Northeast Ohio residents who underwent SARS-CoV-2 screening.

## Methods

### Development of hypothesized causal mechanisms

**Fig 1:**
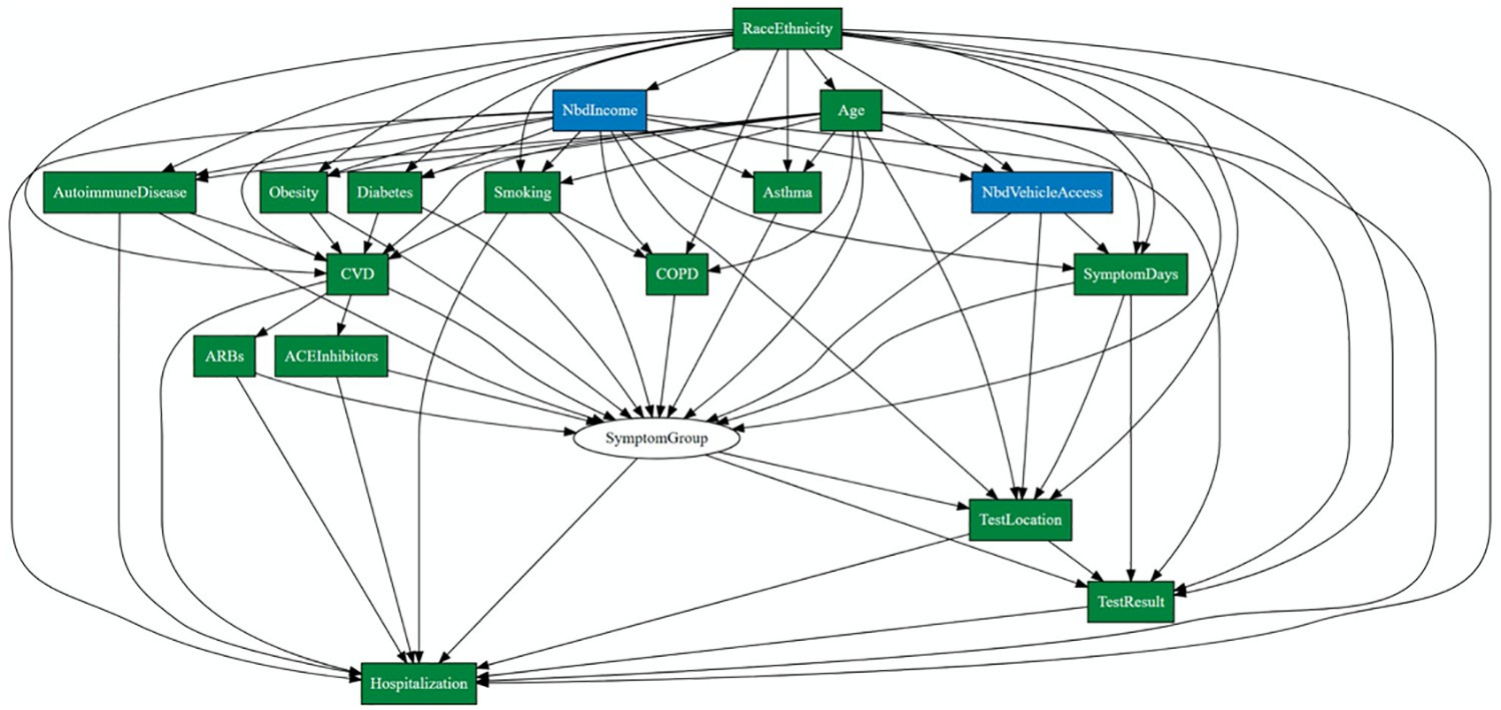
Hypothesized mechanistic model for racial, ethnic and socioeconomic mechanisms of COVID-19 testing location, test positivity and hospitalization.

We conducted a series of 13 weekly videoconferences to construct hypothesized mechanisms linking race/ethnicity and neighborhood income to i) presentation to the emergency department (ED) as opposed to an outpatient testing location for SARS-CoV-2 screening, ii) SARS-CoV-2 test positivity, and iii) COVID-19 hospitalization among those who screened positive for the SARS-Cov-2 virus. We identified several intersecting hypotheses supported by prior literature, and assembled these into a directed acyclic graph (see Fig 1). We detail below the rationale for each of these hypotheses.

We hypothesized that racial, ethnic, and income-related differences in obesity rates [21], asthma [22], chronic obstructive pulmonary disease (COPD) [23–25], type 2 diabetes mellitus (T2DM) [26–29], and other comorbid conditions [30] may result in differences in the extent and severity of COVID-19-related symptom patterns. The Cleveland Clinic Health System (CCHS) collects information on whether or not patients referred for testing have the following symptoms: cough, fever, flu-like symptoms, shortness of breath, diarrhea, loss of appetite, and vomiting. Rather than model individual symptoms, we sought to characterize patterns of co-occurring symptoms. We conducted a sub-analysis which sought to i) identify groups (classes) of patients with similar symptom profiles; ii) characterize the distribution of these symptom classes as a function of sociodemographic and comorbid characteristics (see “Symptom Class” in Fig 1), and iii) relate the derived symptom classes to the likelihood of testing occurring in the ED, likelihood of test positivity, and likelihood of hospitalization. Details on the derivation of these symptom classes are provided below, under Statistical Methods.

Smoking is disproportionately prevalent in disadvantaged communities [31,32], and the prevalence of smoking decreases in older populations. [33] Smoking by itself is associated with COVID-19 disease progression [34], and is a primary risk factor for COPD [35]. It thus represents a potential mechanism to which any observed variation in symptom patterns across the socioeconomic spectrum might be attributable.

Lack of vehicle ownership, whether due to urbanized living environments, neighborhood disadvantage or racial/ethnic disparities in employment, presents a significant barrier to timely outpatient testing utilization. Health care utilization, in general, is reduced among disadvantaged populations due to lack of financial resources, distrust, lack of time due to other responsibilities, lack of transportation, and other factors [36–39]. Therefore, neighborhood-level median income and vehicle ownership rates (defined as the percentage of households within a census tract owning at least one vehicle) may be related to the likelihood that a patient presents to the ED (vs. all other settings) for screening; as well as the nature, duration and severity of symptoms at the time of testing.

### Data sources and inclusion criteria

The Cleveland Clinic COVID-19 registry, approved by the Cleveland Clinic Institutional Review Board, was designed to serve as a large case series study of the clinical characteristics and outcomes of all CCHS patients who are referred for COVID-19 testing. This study population is identified daily through built-in electronic health record (EHR) queries and linked to patients’ demographic, clinical and residential characteristics through the EHR. We included every adult patient who resided in one of 17 Northeast Ohio Counties and who was referred for testing from the registry’s inception on March 17, 2020 through April 15, 2020. We excluded patients who did not receive a test or whose test results were deemed erroneous. We also excluded patients whose symptoms were not documented. Addresses of residence for patients in the registry were mapped to U.S. Census’ Geographic Identifiers (GEOIDs). We extracted from the 2018 American Community Survey neighborhood-level characteristics (such as median income and percent of households owning one or more vehicles) corresponding to census tracts embedded within these GEOIDS using the R packages sociome and tidycensus [40,41].

### Statistical methods

We developed regression models for each variable depicted **in** Fig 1. For each given variable in the network, we included all pre-specified parent nodes as predictors. Continuous and dichotomous variables were modeled using multivariable linear regression and multivariable logistic regression, respectively. Duration of symptoms was modeled using proportional odds logistic regression, with the following ordered levels: asymptomatic, or symptoms of 0–2 days, 3–7 days, 1–2 weeks, and >2 weeks duration. Neighborhood vehicle access, defined for each patient as the proportion of households in their census tract with 1 or more vehicle, was modeled using quasibinomial regression [42], a technique for modeling proportions that are not necessarily expressed as counts of events out of a total number of trials. Multinomial logistic regression was used to model smoking status (non-smoker, current smoker, former smoker, unknown smoking status).

Latent class analysis [43,44]—a method for empirically deriving homogenous groups of patients with respect to a set of binary indicators—was used to derive symptom classes. We estimated, using Mplus software version 8.4 [45], and compared solutions of up to 10 classes. We chose the solution for the number of classes that optimized model goodness of fit criteria (e.g., Bayesian Information Criteria [BIC]; entropy; and sequential likelihood ratio tests [LRTs]). Resulting classes were ordered with respect to increasing incidence of test positivity. Class membership was summarized across age, neighborhood income, race/ethnicity, sex, comorbidity status (set of clinical diagnoses and treatments depicted in Fig 1) and duration of symptoms.

We used quadratic polynomial terms for age and neighborhood income. For models including age, race/ethnicity and neighborhood income as predictor variables, we considered two- and three-way interactions among these variables. Interactions were removed from those models in an iterative manner, beginning with the 3-way interaction, when they did not meaningfully improve model goodness-of-fit criteria (e.g., residual deviance); we chose this approach instead of one strictly driven by statistical significance criteria because our sample size enabled detection of relatively minor interaction effects.

We used the RStudio Integrated Development Environment [46], running R version 3.5.0 (2018-04-23) [47] for all analyses, unless otherwise noted above. A reproducible analysis workflow, including code, analytic datasets and automated statistical reporting were developed using the *projects* and *RMarkdown* R packages [48,49].

## Results

A total of 20392 patients were accrued into the Cleveland Clinic COVID-19 registry over the study period, of whom 14887 were documented as residing in Northeast Ohio. Of these, we removed 36 patients for whom a test was ordered but not performed, 66 additional patients for whom nasopharyngeal test results were deemed erroneous and 1885 additional patients whose symptom data were not recorded (total of 13.3% removed).

**Fig 2:**
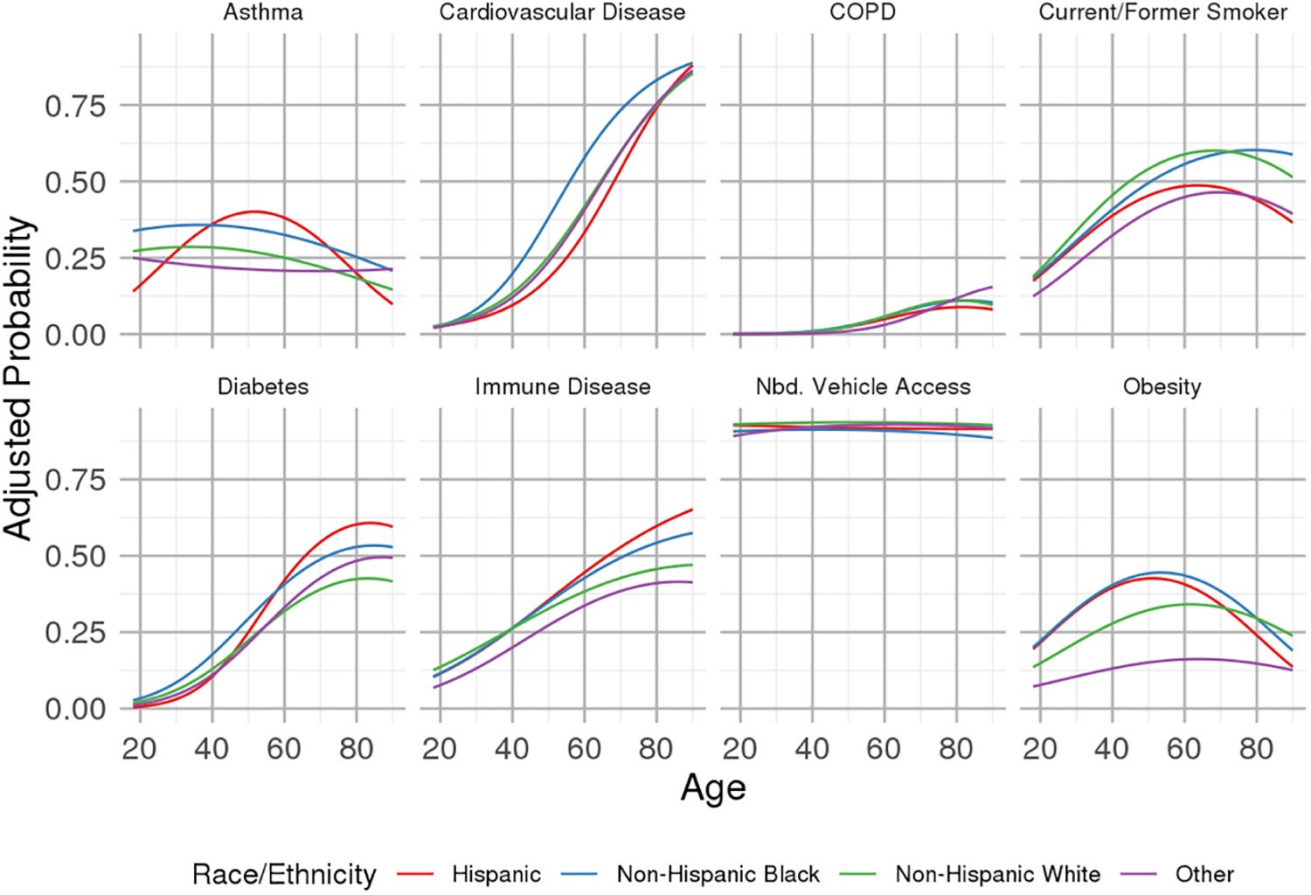
Prevalence of selected comorbid conditions and proportion of households in patients’ respective neighborhood of residence owning at least one vehicle, as a function of age, for groups of patients defined according to race and ethnicity. Estimates adjusted to the observed median neighborhood income of $69,324. Estimates for cardiovascular disease adjusted to modal values of other predictors in the model, i.e., non-diabetic, non-obese, no autoimmune disease, and no documented smoking history. Similarly, estimates for COPD adjusted to no documented smoking history.

**Fig 3:**
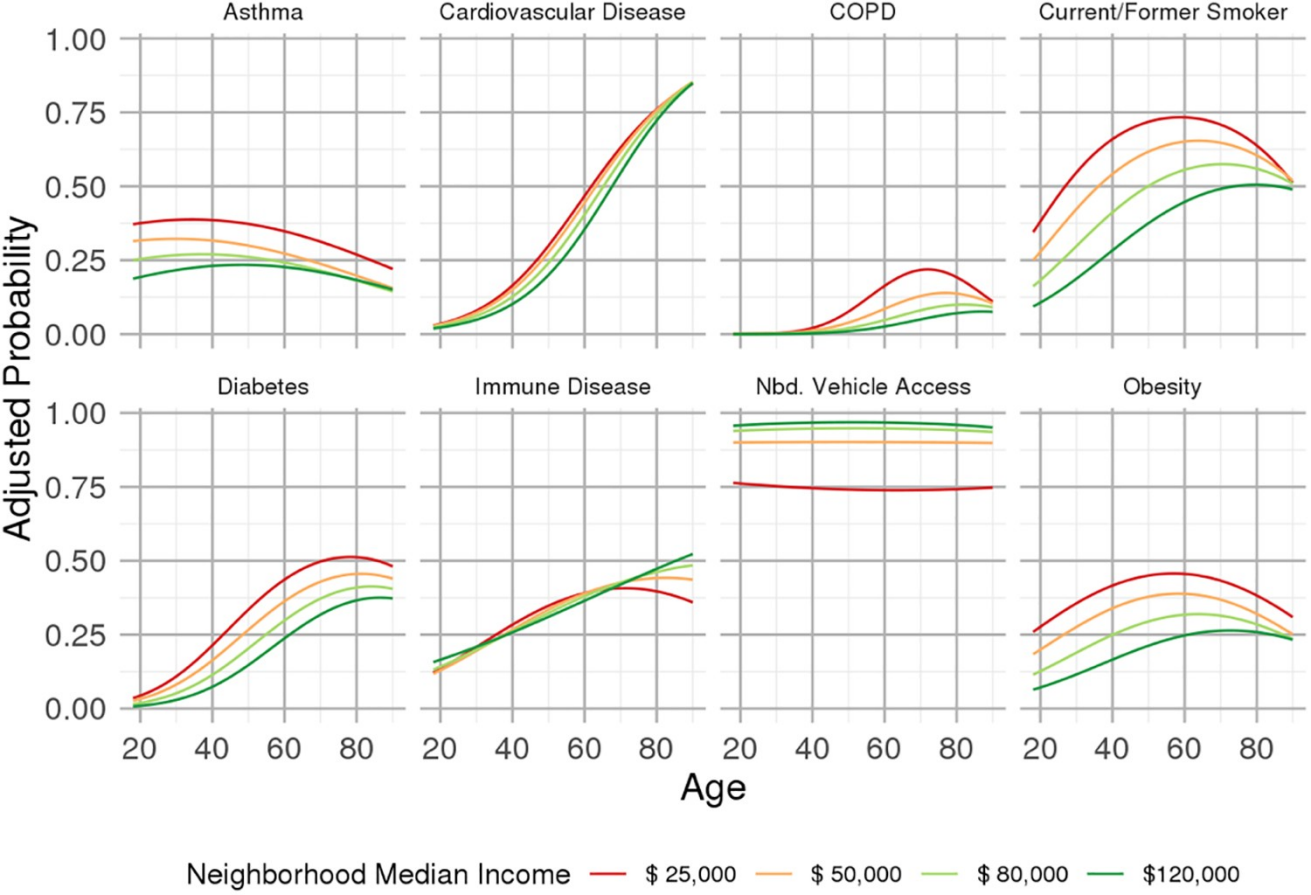
Prevalence of selected comorbid conditions and proportion of households in patients’ respective neighborhood of residence owning at least one vehicle, as a function of age, for groups of patients defined according to selected levels of median neighborhood income. Estimates adjusted to the modal race/ethnicity (non-Hispanic White). Estimates for cardiovascular disease adjusted to modal values of other predictors in the model, i.e., non-diabetic, non-obese, no autoimmune disease, and no documented smoking history. Similarly, estimates for COPD adjusted to no documented smoking history.

Within the patients who met study inclusion criteria, age distributions were comparable between non-Hispanic Black and non-Hispanic White populations (median [first quartile, third quartile] of 52 [37, 65] and 51 [36, 65] years, respectively). Hispanic patients and patients of other racial or ethnic backgrounds tended to be younger (43 [31, 57] and 42 [31, 57] years, respectively)). Sociodemographic and comorbid disease characteristics are summarized across levels of neighborhood median income in Table 1: Patients from lower-income communities were disproportionately non-Hispanic Black and/or Hispanic (see S1 File for distributions of neighborhood median income by race) and had disproportionately higher prevalence of T2DM, obesity, smoking, asthma, cardiovascular disease (CVD) and COPD. Vehicle ownership rates in patients’ neighborhoods of residence were not related to the likelihood of testing in the ED after accounting for neighborhood income and other factors. Curves of the prevalence of comorbid conditions, as well as proportions of households with neighborhood vehicle access, as a function of age are presented by race/ethnicity in Fig 2 and by levels of neighborhood median income in Fig 3. Increasing age was generally related to increased prevalence of CVD, COPD, T2DM and immune disease (see S1 File). Non-Hispanic Black and Hispanic patients exhibited a higher prevalence of CVD and T2DM, as well as increased obesity (based on body mass index recorded in the electronic health record) prevalence in mid-life. Generally, income gradients in these characteristics were more prominent than gradients corresponding to differences in race/ethnicity. Odds ratio estimates from multivariable models that also adjusted for other risk factors as depicted in Fig 1 are provided in S1 File.

**Table 1:** Sociodemographic and clinical characteristics of patients who met study inclusion criteria, by levels of 2018 neighborhood median income corresponding to census tracts of residence in Northeastern Ohio.

	Neighborhood Median Income (USD, thousands)					p-value^2^
	≤$25k N = 389^1^	$25-40k N = 3104^1^	$50-80k N = 4768^1^	$80-120k N = 3485^1^	>$120k N = 1154^1^	
Age (years)	54 (37, 65)	50 (35, 63)	50 (35, 64)	51 (36, 66)	51 (37, 65)	<0.001
Female Sex	230 (59%)	1924 (62%)	2966 (62%)	2131 (61%)	693 (60%)	0.5
Race/Ethnicity						<0.001
Non-Hispanic White	80 (21%)	1274 (41%)	3505 (74%)	2897 (83%)	926 (80%)	
Non-Hispanic Black	267 (69%)	1432 (46%)	783 (16%)	228 (6.5%)	60 (5.2%)	
Hispanic	17 (4.4%)	165 (5.3%)	79 (1.7%)	27 (0.8%)	6 (0.5%)	
Other	25 (6.4%)	233 (7.5%)	401 (8.4%)	333 (9.6%)	162 (14%)	
Autoimmune Disease	136 (37%)	927 (32%)	1447 (32%)	1061 (32%)	359 (33%)	0.4
Diabetes Mellitus	115 (32%)	873 (30%)	1094 (24%)	667 (21%)	190 (18%)	<0.001
Obesity	158 (41%)	1144 (37%)	1414 (30%)	818 (23%)	179 (16%)	<0.001
Current or Former Smoker	227 (58%)	1779 (57%)	2250 (47%)	1402 (40%)	372 (32%)	<0.001
Asthma	129 (35%)	951 (33%)	1186 (27%)	763 (23%)	221 (21%)	<0.001
Cardiovascular Disease	242 (65%)	1672 (56%)	2197 (48%)	1545 (47%)	423 (39%)	<0.001
Chronic Obstructive Pulmonary Disease	70 (19%)	485 (17%)	508 (12%)	265 (8.3%)	63 (6.0%)	<0.001
Neighborhood Vehicle Access (% households)	0.63 (0.49, 0.75)	0.83 (0.76, 0.90)	0.95 (0.91, 0.97)	0.97 (0.94, 0.98)	0.97 (0.94, 0.99)	<0.001

^*1*^ Statistics presented: median (IQR); n (%).

^*2*^ Statistical tests performed: Kruskal-Wallis test; chi-square test of independence.

Regarding the latent class analysis to identify relatively homogeneous groups of patients with respect to their symptoms at testing, the 6-class solution optimized the BIC, and entropy and (0.62) was near optimum relative to solutions involving other numbers of classes. Sequential LRTs all were strongly significant (p<0.001) for all solutions up to the 6-class solution, while the LRT for the 7-class solution relative to the 6-class solution was not significant (p = 0.11). These profiles, along with incidence estimates of SARS-CoV-2 test positivity and a summary of sociodemographic factors are provided in Table 2. Class 1 (n = 2510), which had the lowest incidence of SARS-CoV-2 test positivity at 3.4%, was mostly characterized by cough (incidence: 67%) and shortness of breath (99%) and had the highest incidence of testing in the ED (vs. outpatient locations, 60%). Class 1 patients were older (median [IQR] age: 57 [41, 71] years) and had generally more extensive comorbid disease burden than the other classes. Class 2 (n = 3463; 5.4% test positivity) was the largest and generally lower in symptom burden, with moderate incidence of cough (48%) and relatively short duration of symptoms (57% asymptomatic or having symptoms 2 or fewer days). Class 2 had a higher prevalence of non-Hispanic White race/ethnicity (68%), relatively higher neighborhood median income ($71000 [$49000, $95000]) and relatively low rates of presentation to the ED. Class 3 (n = 684; 8.9% test positivity) had a generally higher symptom burden, with particularly higher prevalence of gastrointestinal symptoms (diarrhea, loss of appetite and vomiting) class. Class 3 was largely characteristic of the broader cohort with respect to demographic and clinical factors, and relatively more likely to have been tested in the ED. Class 4 (n = 2955; 11% test positivity) had symptoms most closely matching influenza (cough, fever and flulike symptoms); symptoms were relatively prolonged in this relatively younger class. Class 5 (n = 2593; 16% test positivity) was similar to Class 2, except that the incidence of cough was higher (90%) and accompanied by fatigue (100%) and fever (55%), and Class 2 patients had over twice the rate of test positivity (5.7% and 16% test positivity for Classes 2 and 5, respectively). Class 6 (n = 695; 23% test positivity) had the highest overall symptom burden—all documented symptoms had incidences of at least 59%—and also had the longest duration of symptoms (31% with symptoms for >1 week). These patients were disproportionately non-Hispanic Black (27%, vs. 21% in the overall sample), were more likely to present to the ED (52%), the most likely to have tested positive (23%) and the most likely to have required hospitalization (11% vs. 5.7% for Class 5, which had the next highest test positivity and hospitalization rates).

**Table 2:** Sociodemographic and clinical characteristics; and COVID-19 screening and clinical outcomes; stratified by symptom class. Patients were assigned to symptom classes based on their modal estimated probability of class membership derived from the latent class model.

	Symptom Class						p-value^2^
	Class 1 N = 2510^1^	Class 2 N = 3463^1^	Class 3 N = 684^1^	Class 4 N = 2955^1^	Class 5 N = 2593^1^	Class 6 N = 695^1^	
COVID-19-Related Symptoms							
Cough	1686 (67%)	1659 (48%)	101 (15%)	2738 (93%)	2343 (90%)	677 (97%)	<0.001
Fever	69 (2.7%)	793 (23%)	246 (36%)	1670 (57%)	1419 (55%)	408 (59%)	<0.001
Fatigue	487 (19%)	173 (5.0%)	296 (43%)	0 (0%)	2593 (100%)	455 (65%)	<0.001
Flulike Symptoms	0 (0%)	105 (3.0%)	171 (25%)	2593 (88%)	2020 (78%)	629 (91%)	<0.001
Shortness of Breath	2489 (99%)	0 (0%)	120 (18%)	1591 (54%)	1283 (49%)	490 (71%)	<0.001
Diarrhea	226 (9.0%)	284 (8.2%)	428 (63%)	465 (16%)	382 (15%)	529 (76%)	<0.001
Loss of Appetite	89 (3.9%)	0 (0%)	329 (53%)	140 (5.3%)	360 (16%)	425 (66%)	<0.001
Vomiting	79 (3.1%)	105 (3.0%)	410 (60%)	59 (2.0%)	0 (0%)	467 (67%)	<0.001
Duration of Symptoms							<0.001
Asymptomatic	54 (2.4%)	79 (2.9%)	7 (1.1%)	39 (1.4%)	35 (1.4%)	5 (0.8%)	
0–2 Days	994 (45%)	1474 (54%)	314 (49%)	897 (33%)	717 (30%)	162 (24%)	
3–7 Days	685 (31%)	754 (27%)	222 (35%)	1170 (43%)	1007 (41%)	294 (44%)	
1–2 Weeks	251 (11%)	239 (8.7%)	57 (8.9%)	373 (14%)	385 (16%)	124 (19%)	
>2 Weeks	237 (11%)	203 (7.4%)	37 (5.8%)	252 (9.2%)	285 (12%)	81 (12%)	
Sociodemographic and Comorbid Characteristics							
Age (years)	57 (41, 71)	50 (35, 66)	52 (35, 68)	46 (34, 60)	49 (36, 63)	49 (36, 60)	<0.001
Female Sex	1515 (60%)	2054 (59%)	436 (64%)	1859 (63%)	1624 (63%)	456 (66%)	0.002
Race/Ethnicity							<0.001
Non-Hispanic White	1627 (65%)	2342 (68%)	453 (66%)	1983 (67%)	1833 (71%)	444 (64%)	
Non-Hispanic Black	703 (28%)	721 (21%)	170 (25%)	579 (20%)	408 (16%)	189 (27%)	
Hispanic	53 (2.1%)	68 (2.0%)	18 (2.6%)	83 (2.8%)	56 (2.2%)	16 (2.3%)	
Other	127 (5.1%)	332 (9.6%)	43 (6.3%)	310 (10%)	296 (11%)	46 (6.6%)	
Neighborhood Median Income ($K)	64 (43, 88)	71 (49, 95)	69 (46, 88)	69 (48, 92)	74 (53, 95)	66 (44, 88)	<0.001
Autoimmune Disease	838 (35%)	1042 (32%)	231 (35%)	824 (29%)	775 (31%)	220 (33%)	<0.001
Diabetes Mellitus	738 (31%)	724 (23%)	193 (30%)	582 (21%)	546 (22%)	156 (24%)	<0.001
Obesity	896 (36%)	811 (23%)	221 (32%)	804 (27%)	732 (28%)	249 (36%)	<0.001
Current or Former Smoker	1423 (57%)	1445 (42%)	316 (46%)	1331 (45%)	1153 (44%)	362 (52%)	<0.001
Asthma	831 (35%)	621 (19%)	128 (20%)	790 (28%)	676 (27%)	204 (31%)	<0.001
Cardiovascular Disease	1543 (64%)	1552 (48%)	353 (54%)	1178 (42%)	1109 (45%)	344 (52%)	<0.001
Chronic Obstructive Pulmonary Disease	576 (25%)	227 (7.2%)	58 (9.3%)	233 (8.5%)	226 (9.4%)	71 (11%)	<0.001
Neighborhood Vehicle Access (% households)	0.93 (0.85, 0.97)	0.94 (0.87, 0.97)	0.94 (0.86, 0.97)	0.94 (0.87, 0.97)	0.95 (0.89, 0.97)	0.93 (0.84, 0.97)	<0.001
COVID-19 Outcomes							
Testing in Emergency Department	1514 (60%)	1198 (35%)	378 (55%)	1125 (38%)	1022 (39%)	364 (52%)	<0.001
Test Positivity	86 (3.4%)	199 (5.7%)	61 (8.9%)	315 (11%)	423 (16%)	163 (23%)	<0.001
Hospitalization	42 (1.7%)	42 (1.2%)	31 (4.5%)	63 (2.1%)	149 (5.7%)	77 (11%)	<0.001

^*1*^ Statistics presented: n (%).

^*2*^ Statistical tests performed: chi-square test of independence.

**Fig 4:**
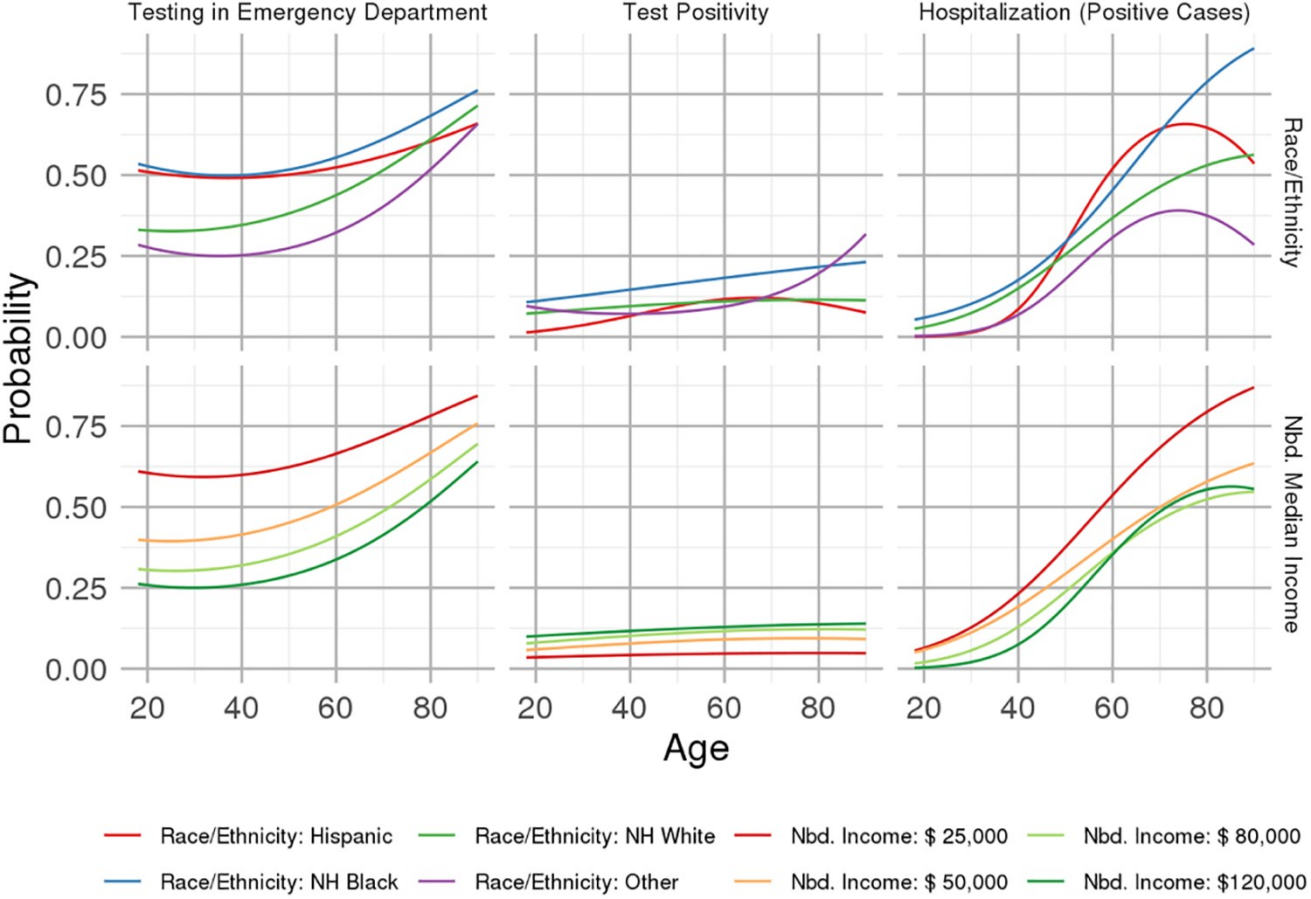
Incidence of testing in the ED (as opposed to any other location), COVID-19 test positivity and hospitalization (among those testing positive for COVID-19), as a function of age, for categories of patients defined according to race/ethnicity (adjusting for median income) and neighborhood median income (adjusting to the modal race/ethnicity, non-Hispanic White).

Curves of the proportion tested in the ED and test positivity as a function of race/ethnicity and neighborhood income are given in Fig 4, and multivariable odds ratio estimates for these outcomes comparing groups defined by race/ethnicity and levels of median neighborhood income (respectively) are given in S1 File. S1 File provides confidence interval estimates for the predicted probabilities given in Fig 4 for selected age values. The analyzed sample of early-pandemic SARS-CoV-2-screened individuals contained too few positive cases to produce reliably similar curves for hospitalization rates among positive cases. Non-Hispanic Black and Hispanic patients, as well as those from lower-income neighborhoods, were more frequently tested in the ED, with the difference in testing location compared to non-Hispanic White and higher-income neighborhoods (respectively) attenuating slightly among individuals of older age. Those whose symptoms were present for more than a week were less likely to present to the ED. Neighborhood vehicle access was not independently related to presentation to the ED.

Non-Hispanic Black patients were between 1.81 [95% confidence interval: 0.91–3.59] times (at age 20) to 2.37 [1.54–3.65] times (at age 80) more likely to test positive for the SARS-CoV-2 virus, while test positivity was not significantly different across the neighborhood income spectrum. Differences in test positivity remained across the six symptom classes after multivariable adjustment, with the highest risk class (Class 6) being 10.0 [7.4–13.6] times as likely to test positive as the lowest risk class (Class 1).

Among the 1247 patients who tested positive, 404 (32.4%) were hospitalized. Results from the multivariable model for hospitalization were not precise due to the relatively low number of hospitalizations in the sample, although testing in the emergency department (OR: 5.4 [3.9, 7.5]) and cardiovascular disease diagnosis (OR: 2.5 [1.7, 3.8]) were related to increased risk.

## Discussion

This study was aimed at identifying socioeconomic and chronic disease-related processes that may influence access to SARS-CoV-2 testing, test positivity, and COVID-19-related hospitalization among a cohort of Northeast Ohio residents who were tested for SARS-CoV-2. The key findings of this study were that tested patients residing in lower-income neighborhoods, who were more likely to be Non-Hispanic Black and/or Hispanic, i) had a higher prevalence of comorbidities such as T2DM and CVD; ii) were more likely to present to the ED for testing than any other location; and iii) were more likely to be hospitalized for COVID-19. Furthermore, non-Hispanic Blacks were more likely to test positive for COVID-19 compared with all others. The increased likelihood of presenting to the ED for testing among Hispanic and non-Hispanic Black patients (compared to non-Hispanic White patients) was more apparent among younger individuals, and appeared to attenuate in the older populations.

Since the beginning of the pandemic, much has been published on the associations of prevalent comorbidities, such as hypertension, T2DM, obesity, coronary artery disease with elevated COVID-19 incidence and case-fatality [50,51]. Such outcomes-based research has allowed policy-makers and public-health specialists to identify individuals who may benefit the most from social distancing and other preventive measures against COVID-19 and highlighted racial/ethnic and socio-economic disparities in healthcare access, and a possible disproportionate impact of the pandemic on persons from racial and ethnic minority backgrounds. The earliest reports came from New York City, where ecological researchers found a disproportionately higher rate of COVID-19 infection and its associated mortality among residents of the Bronx, a predominantly lower-income, Hispanic- and non-Hispanic Black-inhabited borough compared with Manhattan, a predominantly middle-to-high income, non-Hispanic White-inhabited borough [5]. Similar trends were reported in other urban hotspots and predominantly non-Hispanic Black-inhabited counties across the United States [52]. Identifying the factors that may be at play behind such glaring healthcare disparities is of paramount importance, with the ultimate aim of addressing them to ensure that all individuals, regardless of their socio-economic background are able to benefit from the advancements of medical care.

The adverse effects of social determinants of health are more pronounced in an airborne pandemic such as COVID-19 [53]. Better living facilities that allow for adequate social distancing, adequate access to healthcare, job and income security providing opportunities for working from home, and other changes to work status and work environments all may impact an individual’s risk for contracting and dying from the disease. Historically, non-Hispanic Black and Hispanic persons have not enjoyed a full share of these opportunities in the social hierarchy of American society. Our study, of a large healthcare system serving a socio-economically diverse population, allowed us to explore individual level differences in race, income, vehicle access and other social determinants and their impact on COVID-19 outcomes. This is in contrast to other ecological studies [5], which have measured associations at population aggregate levels such as counties and boroughs. The finding that socioeconomically disadvantaged, Non-Hispanic Black and Hispanic groups were more likely to present to the ED for testing than any other location may be due to downstream effects of policy that, despite measures such as the Fair Housing Act, have resulted in generatively entrenched, multi-generational disparities that have led to concentrated poverty and hardship. Coincident with lack of access and means for accessing outpatient testing facilities are a lack of healthcare literacy, inadequate of primary care services in these neighborhoods, concerns, perceptions and fears of social biases associated with the healthcare system writ large, and possible implicit bias within the system connecting those who are in need of testing to those who provide testing. Despite the implementation of the Affordable Care Act in Ohio, health insurance inevitably remains tied with employment. It is likely that given the high proportion of non-Hispanic Blacks and Hispanics employed in lower-paying, contingent jobs, the economic burden of the pandemic disproportionately affects lower-income communities and racial and ethnic minorities in particular, rendering workers uninsured and with few options other than emergency care. Recognition of these disparities offers a unique opportunity to target interventions that help vulnerable populations in high need areas, including providing transportation to clinics, food and meal delivery, connections to primary care services, and peer support. Recently published research from our region, that examined patients who utilized a physician-staffed telephone hotline with wrap-around social services, suggests that such approaches have promise for improving health access, reaching patients earlier and reducing social health disparities.

The high test-positivity rate in the ED is likely due (at least in part) to severe regional constraints on the availability of testing early in the pandemic, even to persons presenting to primary care with some symptoms. Nonetheless, the findings that racial/ethnic and economic disparities in testing access, test positivity, as well as hospitalization from COVID-19 were more pronounced in younger individuals compared with older individuals likely reflect a combination of phenomena. First, in a younger population with fewer comorbidities, effects associated with social and economic deprivation may be more pronounced, whereas adverse selection processes (such as premature mortality) may lead to attenuation of observed differences in older individuals. Second, younger persons may have had a higher likelihood of exposure due to working front line jobs and not being designated as an ‘at risk’ group. Third, research suggests that persons in lower-income communities exhibit signs of accelerated or premature aging, inflammation and immune dysregulation; these vulnerabilities are likely to translate into differences in the severity of COVID-19 disease.

The finding that individuals with a higher prevalence of disease symptoms (class 6) were more likely to be Non-Hispanic Blacks and/or Hispanic, and, in turn, were more likely to test positive and be hospitalized, is especially concerning. What we cannot describe from these data is the degree to which better access to earlier screening and care might have reduced this disparity. Thus, further understanding of processes both endogenous and exogenous to the healthcare system that, coalesce to produce racial and economic inequalities in COVID-19 outcomes—and policy changes to ameliorate these inequalities—is critically needed.

## Supporting information

S1 File S1 File contains details on additional supplementary tables and figures and can be found online. (DOCX)

## References

[1] Pan D, Sze S, Minhas JS, et al. The impact of ethnicity on clinical outcomes in COVID-19: A systematic review. *EClinicalMedicine*. 2020;23:100404. doi: 10.1016/j.eclinm.2020.100404 32632416 PMC7267805

[2] Lassale C, Gaye B, Hamer M, Gale CR, Batty GD. Ethnic Disparities in Hospitalisation for COVID-19 in England: The Role of Socioeconomic Factors, Mental Health, and Inflammatory and Pro-inflammatory Factors in a Community-based Cohort Study. *Brain Behav Immun*. Published online 2020. https://www.sciencedirect.com/science/article/pii/S0889159120311016. 10.1016/j.bbi.2020.05.074 PMC7263214 32497776

[3] Garg S. Hospitalization rates and characteristics of patients hospitalized with laboratory-confirmed coronavirus disease 2019—COVID-NET, 14 States, March 1–30, 2020. *MMWR Morb Mortal Wkly Rep*. 2020;69. https://www.cdc.gov/mmwr/volumes/69/wr/mm6915e3.htm?s%20cid=mm6915e3%20w. doi: 10.15585/mmwr.mm6915e3 32298251 PMC7755063

[4] Williamson EJ, Walker AJ, Bhaskaran K, et al. Factors associated with COVID-19-related death using OpenSAFELY. *Nature*. 2020;584(7821):430–436. doi: 10.1038/s41586-020-2521-4 32640463 PMC7611074

[5] Wadhera RK, Wadhera P, Gaba P, et al. Variation in COVID-19 Hospitalizations and Deaths Across New York City Boroughs. *JAMA*. 2020;323(21):2192–2195. doi: 10.1001/jama.2020.7197 32347898 PMC7191469

[6] Adhikari S, Pantaleo NP, Feldman JM, Ogedegbe O, Thorpe L, Troxel AB. Assessment of Community-Level Disparities in Coronavirus Disease 2019 (COVID-19) Infections and Deaths in Large US Metropolitan Areas. *JAMA Netw Open*. 2020;3(7):e2016938. doi: 10.1001/jamanetworkopen.2020.16938 32721027 PMC7388025

[7] Kirby T. Evidence mounts on the disproportionate effect of COVID-19 on ethnic minorities. *Lancet Respir Med*. 2020;8(6):547–548. doi: 10.1016/S2213-2600(20)30228-9 32401711 PMC7211498

[8] de Lusignan S, Dorward J, Correa A, et al. Risk factors for SARS-CoV-2 among patients in the Oxford Royal College of General Practitioners Research and Surveillance Centre primary care network: a cross-sectional study. *Lancet Infect Dis*. 2020;20(9):1034–1042. doi: 10.1016/S1473-3099(20)30371-6 32422204 PMC7228715

[9] Burström B, Tao W. Social determinants of health and inequalities in COVID-19. *Eur J Public Health*. 2020;30(4):617–618. doi: 10.1093/eurpub/ckaa095 32638998 PMC7454505

[10] Rogers TN, Rogers CR, VanSant-Webb E, Gu LY, Yan B, Qeadan F. Racial Disparities in COVID-19 Mortality Among Essential Workers in the United States. *World Med Health Policy*. Published online August 5, 2020. doi: 10.1002/wmh3.358 32837779 PMC7436547

[11] Moore JT, Ricaldi JN, Rose CE, et al. Disparities in Incidence of COVID-19 Among Underrepresented Racial/Ethnic Groups in Counties Identified as Hotspots During June 5–18, 2020–22 States, February-June 2020. *MMWR Morb Mortal Wkly Rep*. 2020;69(33):1122–1126. doi: 10.15585/mmwr.mm6933e1 32817602 PMC7439982

[12] U.S. Centers for Disease Control and Prevention. COVID-19 Hospitalization and Death by Race/Ethnicity. Coronavirus Disease 2019. Accessed August 21, 2020. https://www.cdc.gov/coronavirus/2019-ncov/covid-data/investigations-discovery/hospitalization-death-by-race-ethnicity.html.

[13] White C, Nafilyan V. Coronavirus (COVID-19) related deaths by ethnic group, England and Wales: 2 March 2020 to 10 April 2020. Office of National Statistics. Accessed August 21, 2020. https://www.ons.gov.uk/peoplepopulationandcommunity/birthsdeathsandmarriages/deaths/articles/coronavirusrelateddeathsbyethnicgroupenglandandwales/2march2020to10april2020.

[14] McLaren J. Racial Disparity in COVID-19 Deaths: Seeking Economic Roots with Census data. Published online 2020. doi: 10.3386/w27407

[15] Yancy CW. COVID-19 and African Americans. *JAMA*. 2020;323(19):1891–1892. doi: 10.1001/jama.2020.6548 32293639

[16] Webb Hooper M, Nápoles AM, Pérez-Stable EJ. COVID-19 and Racial/Ethnic Disparities. *JAMA*. 2020;323(24):2466–2467. doi: 10.1001/jama.2020.8598 32391864 PMC9310097

[17] Laurencin CT, McClinton A. The COVID-19 Pandemic: a Call to Action to Identify and Address Racial and Ethnic Disparities. *J Racial Ethn Health Disparities*. 2020;7(3):398–402. doi: 10.1007/s40615-020-00756-0 32306369 PMC7166096

[18] van Dorn A, Cooney RE, Sabin ML. COVID-19 exacerbating inequalities in the US. *Lancet*. 2020;395(10232):1243. doi: 10.1016/S0140-6736(20)30893-X 32305087 PMC7162639

[19] Chowkwanyun M, Reed AL Jr. Racial health disparities and Covid-19—caution and context. *N Engl J Med*. Published online 2020. https://www.nejm.org/doi/full/10.1056/NEJMp2012910. 10.1056/NEJMp2012910 32374952

[20] Abrams EM, Szefler SJ. COVID-19 and the impact of social determinants of health. *The Lancet Respiratory Medicine*. Published online 2020. https://www.ncbi.nlm.nih.gov/pmc/articles/pmc7234789/. doi: 10.1016/S2213-2600(20)30234-4 32437646 PMC7234789

[21] Arroyo-Johnson C, Mincey KD. Obesity Epidemiology Worldwide. *Gastroenterol Clin North Am*. 2016;45(4):571–579. doi: 10.1016/j.gtc.2016.07.012 27837773 PMC5599163

[22] Forno E, Celedon JC. Asthma and ethnic minorities: socioeconomic status and beyond. *Curr Opin Allergy Clin Immunol*. 2009;9(2):154–160. doi: 10.1097/aci.0b013e3283292207 19326508 PMC3920741

[23] Mamary AJ, Stewart JI, Kinney GL, et al. Race and Gender Disparities are Evident in COPD Underdiagnoses Across all Severities of Measured Airflow Obstruction. *Int J Chron Obstruct Pulmon Dis*. 2018;5(3):177–184. doi: 10.15326/jcopdf.5.3.2017.0145 30584581 PMC6296789

[24] Eisner MD, Blanc PD, Omachi TA, et al. Socioeconomic status, race and COPD health outcomes. *J Epidemiol Community Health*. 2011;65(1):26–34. doi: 10.1136/jech.2009.089722 19854747 PMC3017471

[25] Gershon AS, Dolmage TE, Stephenson A, Jackson B. Chronic obstructive pulmonary disease and socioeconomic status: a systematic review. *COPD*. 2012;9(3):216–226. doi: 10.3109/15412555.2011.648030 22497534

[26] Spanakis EK, Golden SH. Race/ethnic difference in diabetes and diabetic complications. *Curr Diab Rep*. 2013;13(6):814–823. doi: 10.1007/s11892-013-0421-9 24037313 PMC3830901

[27] Signorello LB, Schlundt DG, Cohen SS, et al. Comparing diabetes prevalence between African Americans and Whites of similar socioeconomic status. *Am J Public Health*. 2007;97(12):2260–2267. doi: 10.2105/AJPH.2006.094482 17971557 PMC2089102

[28] Campbell JA, Walker RJ, Smalls BL, Egede LE. Glucose control in diabetes: the impact of racial differences on monitoring and outcomes. *Endocrine*. 2012;42(3):471–482. doi: 10.1007/s12020-012-9744-6 22815042 PMC3779599

[29] Saydah S, Lochner K. Socioeconomic status and risk of diabetes-related mortality in the U.S. *Public Health Rep*. 2010;125(3):377–388. doi: 10.1177/003335491012500306 20433032 PMC2848262

[30] Akinyemiju T, Jha M, Moore JX, Pisu M. Disparities in the prevalence of comorbidities among US adults by state Medicaid expansion status. *Prev Med*. 2016;88:196–202. doi: 10.1016/j.ypmed.2016.04.009 27095325 PMC4902718

[31] Hitchman SC, Fong GT, Zanna MP, Thrasher JF, Chung-Hall J, Siahpush M. Socioeconomic status and smokers’ number of smoking friends: findings from the International Tobacco Control (ITC) Four Country Survey. *Drug Alcohol Depend*. 2014;143:158–166. doi: 10.1016/j.drugalcdep.2014.07.019 25156228 PMC4209373

[32] Hiscock R, Bauld L, Amos A, Fidler JA, Munafò M. Socioeconomic status and smoking: a review. *Ann N Y Acad Sci*. 2012;1248:107–123. doi: 10.1111/j.1749-6632.2011.06202.x 22092035

[33] Centers for Disease Control and Prevention (CDC). Vital signs: current cigarette smoking among adults aged ≥18 years with mental illness—United States, 2009–2011. *MMWR Morb Mortal Wkly Rep*. 2013;62(5):81–87. 23388551 PMC4604817

[34] Patanavanich R, Glantz SA. Smoking Is Associated With COVID-19 Progression: A Meta-analysis. *Nicotine Tob Res*. 2020;22(9):1653–1656. doi: 10.1093/ntr/ntaa082 32399563 PMC7239135

[35] Forey BA, Thornton AJ, Lee PN. Systematic review with meta-analysis of the epidemiological evidence relating smoking to COPD, chronic bronchitis and emphysema. *BMC Pulm Med*. 2011;11:36. doi: 10.1186/1471-2466-11-36 21672193 PMC3128042

[36] Hussein M, Diez Roux AV, Field RI. Neighborhood Socioeconomic Status and Primary Health Care: Usual Points of Access and Temporal Trends in a Major US Urban Area. *J Urban Health*. 2016;93(6):1027–1045. doi: 10.1007/s11524-016-0085-2 27718048 PMC5126022

[37] Riley WJ. Health disparities: gaps in access, quality and affordability of medical care. *Trans Am Clin Climatol Assoc*. 2012;123:167–172; discussion 172–4. 23303983 PMC3540621

[38] Syed ST, Gerber BS, Sharp LK. Traveling towards disease: transportation barriers to health care access. *J Community Health*. 2013;38(5):976–993. doi: 10.1007/s10900-013-9681-1 23543372 PMC4265215

[39] Kangovi S, Barg FK, Carter T, Long JA, Shannon R, Grande D. Understanding why patients of low socioeconomic status prefer hospitals over ambulatory care. *Health Aff*. 2013;32(7):1196–1203. doi: 10.1377/hlthaff.2012.0825 23836734

[40] Krieger NI, Wang C, Dalton JE, Perzynski AT. *Sociome: Helping Researchers to Operationalize Social Determinants of Health Data*. *R Package Version 1.4.0*. *URL*: https://Github.Com/NikKrieger/Sociome.; 2020.

[41] Walker KE. *Tidycensus: Load US Census Boundary and Attribute Data as ‘Tidyverse’and ‘Sf’-Ready Data Frames*.; 2018.

[42] Faraway JJ. *Extending the Linear Model with R*: *Generalized Linear*, *Mixed Effects and Nonparametric Regression Models*, Second Edition. CRC Press; 2016.

[43] Ghosh JK. Latent Class and Latent Transition Analysis: With Applications in the Social, Behavioral, and Health Sciences by Linda M. Collins, Stephanie T. Lanza. *International Statistical Review*. 2010;78(3):449–450. doi: 10.1111/j.1751-5823.2010.00122_5.x

[44] Lazarsfeld PF, Henry NW. *Latent Structure Analysis*. Houghton Mifflin Co.; 1968.

[45] Muthén LK, Muthén B. Mplus. *The comprehensive modelling program for applied researchers*: *user’s guide*. 2019;5. http://www.statmodel.com/virg_nov_course.shtml.

[46] Team R, Others. RStudio: integrated development for R. RStudio, Inc, Boston, MA *URL* http://www.rstudio.com. 2015;42:14.

[47] R Core Team. R: A Language and Environment for Statistical Computing. Published online 2020. https://www.R-project.org/.

[48] Krieger NI, Perzynski AT, Dalton JE. Facilitating reproducible project management and manuscript development in team science: The projects R package. *PLoS One*. 2019;14(7):e0212390. doi: 10.1371/journal.pone.0212390 31356588 PMC6662995

[49] Allaire J, Xie Y, McPherson J, et al. rmarkdown: Dynamic Documents for R. *R package version*. 2018;1(11).

[50] Grasselli G, Zangrillo A, Zanella A, et al. Baseline Characteristics and Outcomes of 1591 Patients Infected With SARS-CoV-2 Admitted to ICUs of the Lombardy Region, Italy. *JAMA*. 2020;323(16):1574–1581. doi: 10.1001/jama.2020.5394 32250385 PMC7136855

[51] Suleyman G, Fadel RA, Malette KM, et al. Clinical Characteristics and Morbidity Associated With Coronavirus Disease 2019 in a Series of Patients in Metropolitan Detroit. *JAMA Netw Open*. 2020;3(6):e2012270. doi: 10.1001/jamanetworkopen.2020.12270 32543702 PMC7298606

[52] Millett GA, Jones AT, Benkeser D, et al. Assessing differential impacts of COVID-19 on black communities. *Ann Epidemiol*. 2020;47:37–44. doi: 10.1016/j.annepidem.2020.05.003 32419766 PMC7224670

[53] Abrams EM, Szefler SJ. COVID-19 and the impact of social determinants of health. The Lancet Respiratory Medicine. 2020 Jul 1;8(7):659–61. doi: 10.1016/S2213-2600(20)30234-4 32437646 PMC7234789

