# Supplementary material for: Mechanisms of socioeconomic differences in COVID-19 screening and hospitalizations
Source: PLoS One. 2021 Aug 5;16(8):e0255343. doi: 10.1371/journal.pone.0255343 (PMC8341486; doi:10.1371/journal.pone.0255343)
Supplement: S1 File — (DOCX) [file pone.0255343.s001.docx]

# **Supplemental Figure Legend**

**Supplemental Figure 1:** Density plots of neighborhood median income, stratified by race and ethnicity. Median and first and third quartiles within each distribution are indicated by vertical black lines overlaid on the density plots.

# **Supplemental Figure 1**

**
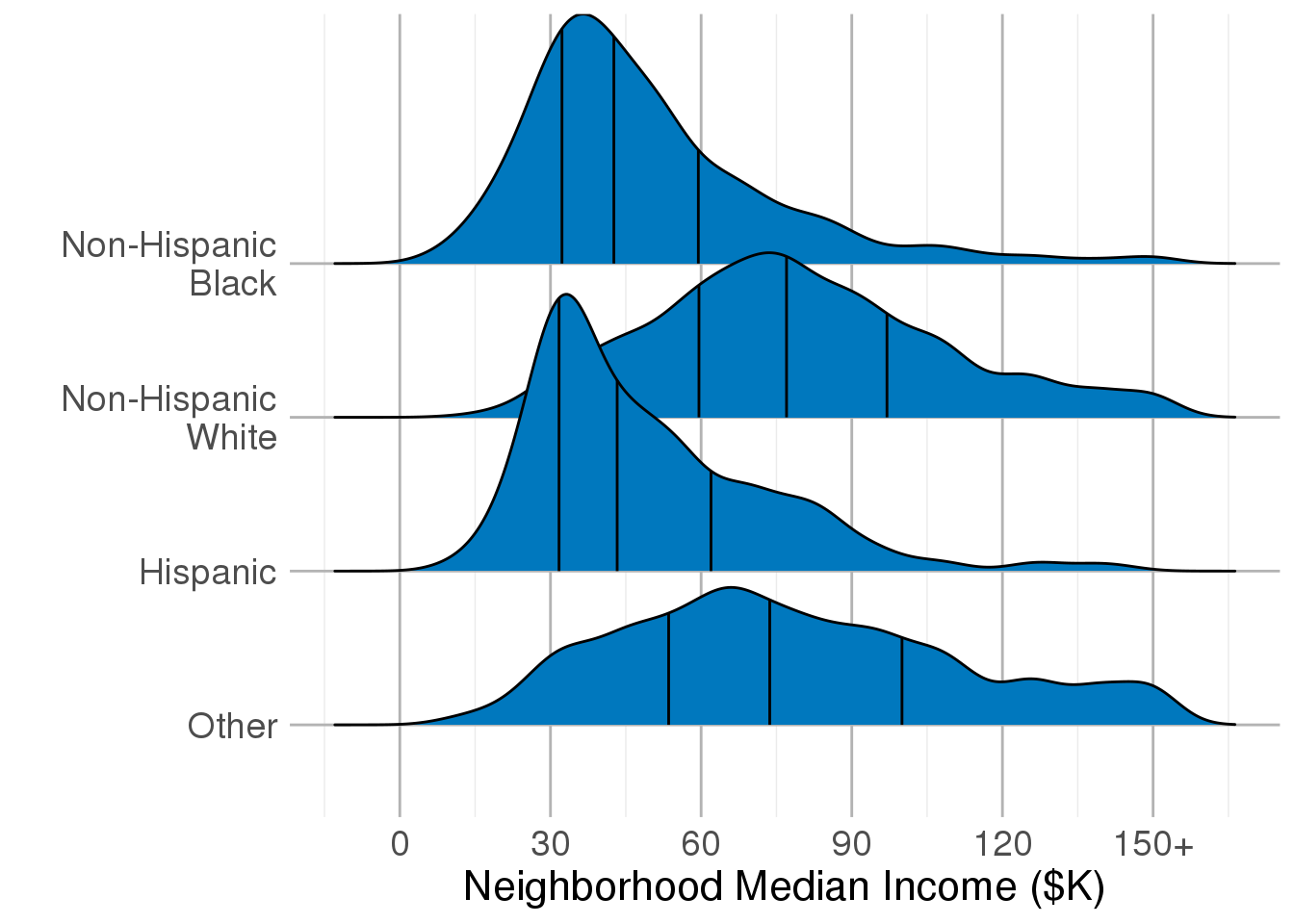
**

#

# **Supplemental Table 1**

Adjusted odds ratios for selected comorbid conditions and proportion of households in patients’ respective neighborhood of residence owning at least one vehicle comparing values of age, derived from respective multivariable logistic regression models. Predictor variables were included in each respective model based on the structure of the hypothesized mechanistic model (see Figure 1).

| **Outcome** | **Age Comparison** | **Odds Ratio [95% CI]** |  | **Outcome** | **Age Comparison** | **Odds Ratio [95% CI]** |
| --- | --- | --- | --- | --- | --- | --- |
| Immune Disease | 40 vs. 20 | 2.61 [2.03, 3.35] |  | Asthma | 40 vs. 20 | 1.35 [1.08, 1.69] |
|  | 60 vs. 20 | 5.1 [3.67, 7.07] |  |  | 60 vs. 20 | 1.3 [0.986, 1.73] |
|  | 60 vs. 40 | 1.95 [1.76, 2.16] |  |  | 60 vs. 40 | 0.963 [0.875, 1.06] |
|  | 80 vs. 20 | 7.43 [5.47, 10.1] |  |  | 80 vs. 20 | 0.894 [0.671, 1.19] |
|  | 80 vs. 40 | 2.85 [2.27, 3.58] |  |  | 80 vs. 40 | 0.66 [0.503, 0.866] |
|  | 80 vs. 60 | 1.46 [1.21, 1.76] |  |  | 80 vs. 60 | 0.685 [0.551, 0.852] |
| Diabetes | 40 vs. 20 | 9.19 [6.39, 13.2] |  | Cardiovascular Disease | 40 vs. 20 | 5.63 [4.01, 7.92] |
|  | 60 vs. 20 | 37.2 [22.6, 61] |  |  | 60 vs. 20 | 28 [18.3, 43] |
|  | 60 vs. 40 | 4.04 [3.47, 4.71] |  |  | 60 vs. 40 | 4.97 [4.31, 5.74] |
|  | 80 vs. 20 | 66.1 [41.9, 104] |  |  | 80 vs. 20 | 123 [80, 189] |
|  | 80 vs. 40 | 7.2 [5.57, 9.3] |  |  | 80 vs. 40 | 21.8 [14.6, 32.5] |
|  | 80 vs. 60 | 1.78 [1.45, 2.18] |  |  | 80 vs. 60 | 4.39 [3.18, 6.06] |
| Obesity | 40 vs. 20 | 2.15 [1.71, 2.7] |  | COPD | 40 vs. 20 | 31.1 [13, 74.4] |
|  | 60 vs. 20 | 2.59 [1.94, 3.46] |  |  | 60 vs. 20 | 260 [73, 923] |
|  | 60 vs. 40 | 1.21 [1.1, 1.33] |  |  | 60 vs. 40 | 8.35 [5.54, 12.6] |
|  | 80 vs. 20 | 1.76 [1.31, 2.35] |  |  | 80 vs. 20 | 583 [170, >999] |
|  | 80 vs. 40 | 0.818 [0.627, 1.07] |  |  | 80 vs. 40 | 18.8 [11.3, 31.1] |
|  | 80 vs. 60 | 0.678 [0.547, 0.839] |  |  | 80 vs. 60 | 2.25 [1.68, 3.01] |
| Current/Former Smoker | 40 vs. 20 | 2.9 [2.37, 3.56] |  | Nbd. Vehicle Access | 40 vs. 20 | 1.09 [1.01, 1.16] |
|  | 60 vs. 20 | 4.79 [3.69, 6.23] |  |  | 60 vs. 20 | 1.08 [0.99, 1.18] |
|  | 60 vs. 40 | 1.65 [1.52, 1.8] |  |  | 60 vs. 40 | 0.997 [0.968, 1.03] |
|  | 80 vs. 20 | 4.51 [3.49, 5.82] |  |  | 80 vs. 20 | 0.991 [0.907, 1.08] |
|  | 80 vs. 40 | 1.55 [1.25, 1.93] |  |  | 80 vs. 40 | 0.913 [0.845, 0.986] |
|  | 80 vs. 60 | 0.941 [0.79, 1.12] |  |  | 80 vs. 60 | 0.915 [0.861, 0.973] |

# **Supplemental Table 2**

Adjusted odds ratios for selected comorbid conditions and proportion of households in patients’ respective neighborhood of residence owning at least one vehicle, derived from respective multivariable logistic regression models. Odds ratios for race/ethnicity and neighborhood income are reported conditional on selected age values (20, 40, 60 and 80 years), due to the presence of significant nonlinear interactions for these factors. Predictor variables were included in each respective model based on the structure of the hypothesized mechanistic model (see **Figure 1**). Comparator/reference values for selected risk factors are: non-Hispanic White (for race/ethnicity); $120,000 (for neighborhood median income); asymptomatic (for duration of symptoms); Class 1 (for symptom class); and testing in the outpatient setting (for testing in the ED). Effects for neighborhood vehicle access are reported on a relative basis per increase in 10%

|  | | | | **Age-Specific Adjusted Odds Ratios** | | | |
| --- | --- | --- | --- | --- | --- | --- | --- |
| **Outcome** | **Risk Factor** | **Value** | **Adjusted Odds Ratio** | **At Age 20** | **At Age 40** | **At Age 60** | **At Age 80** |
| Asthma | Race/Ethnicity | Non-Hispanic Black |  | 1.35 [0.92—1.98] | 1.38 [1.15—1.65] | 1.42 [1.18—1.72] | 1.48 [1.08—2.02] |
|  |  | Hispanic |  | 0.54 [0.22—1.34] | 1.51 [0.92—2.48] | 1.96 [1.13—3.41] | 1.17 [0.42—3.27] |
|  |  | Other |  | 0.85 [0.51—1.41] | 0.70 [0.53—0.92] | 0.77 [0.56—1.07] | 1.15 [0.68—1.95] |
|  | Nbd. Income | $25,000 |  | 2.38 [1.28—4.45] | 1.99 [1.30—3.04] | 1.73 [1.11—2.68] | 1.56 [0.89—2.73] |
|  |  | $50,000 |  | 2.34 [1.38—3.96] | 1.86 [1.27—2.73] | 1.54 [1.04—2.28] | 1.33 [0.84—2.09] |
|  |  | $80,000 |  | 1.66 [1.20—2.30] | 1.44 [1.13—1.82] | 1.27 [1.00—1.62] | 1.15 [0.87—1.53] |
| Cardiovascular Disease | Race/Ethnicity | Non-Hispanic Black |  | 0.93 [0.51—1.72] | 1.55 [1.25—1.94] | 1.87 [1.48—2.36] | 1.62 [0.95—2.75] |
|  |  | Hispanic |  | 0.87 [0.20—3.83] | 0.68 [0.35—1.29] | 0.70 [0.36—1.35] | 0.96 [0.22—4.21] |
|  |  | Other |  | 0.84 [0.36—1.95] | 0.90 [0.65—1.23] | 0.97 [0.69—1.36] | 1.05 [0.52—2.12] |
|  | Nbd. Income | $25,000 |  | 1.67 [0.67—4.19] | 1.85 [1.13—3.05] | 1.70 [1.03—2.81] | 1.30 [0.59—2.85] |
|  |  | $50,000 |  | 1.42 [0.67—2.99] | 1.51 [0.96—2.37] | 1.41 [0.90—2.20] | 1.16 [0.67—2.02] |
|  |  | $80,000 |  | 1.20 [0.76—1.90] | 1.24 [0.93—1.64] | 1.19 [0.90—1.57] | 1.07 [0.76—1.52] |
|  | Diabetes |  | 4.37 [3.84—4.97] |  |  |  |  |
|  | Obesity |  | 2.34 [2.10—2.61] |  |  |  |  |
|  | Immune Disease |  | 1.47 [1.32—1.63] |  |  |  |  |
|  | Current/Former Smoker |  | 1.43 [1.30—1.58] |  |  |  |  |
| COPD | Race/Ethnicity | Non-Hispanic Black |  | 2.30 [0.47— 11.21] | 1.28 [0.74— 2.19] | 0.95 [0.72— 1.26] | 0.95 [0.68— 1.32] |
|  |  | Hispanic |  | 2.13 [0.04—106.84] | 1.13 [0.30— 4.32] | 0.79 [0.31— 2.01] | 0.74 [0.24— 2.25] |
|  |  | Other |  | 0.29 [0.01— 14.85] | 0.32 [0.08— 1.34] | 0.49 [0.27— 0.89] | 1.03 [0.55— 1.93] |
|  | Nbd. Income | $25,000 |  | 1.75 [0.12—25.25] | 3.59 [1.22—10.53] | 3.34 [1.66— 6.70] | 1.41 [0.68— 2.96] |
|  |  | $50,000 |  | 2.58 [0.27—25.06] | 2.80 [1.10— 7.14] | 2.24 [1.23— 4.05] | 1.32 [0.71— 2.44] |
|  |  | $80,000 |  | 1.90 [0.46— 7.86] | 1.78 [1.00— 3.18] | 1.51 [1.04— 2.20] | 1.17 [0.79— 1.73] |
|  | Current/Former Smoker |  | 5.84 [4.96—6.88] |  |  |  |  |
| Current/Former Smoker | Race/Ethnicity | Non-Hispanic Black |  | 0.92 [0.63—1.35] | 0.81 [0.69—0.96] | 0.86 [0.72—1.02] | 1.10 [0.85—1.42] |

# **Supplemental Table 3**

Adjusted odds ratios for testing in the ED (as opposed to any other location), COVID-19 test positivity and hospitalization (among those testing positive for COVID-19), derived from respective multivariable logistic regression models. Odds ratios for race/ethnicity and neighborhood income are reported conditional on selected age values (20, 40, 60 and 80 years), due to the presence of significant nonlinear interactions for these factors. Predictor variables were included in each respective model based on the structure of the causal model (see **Figure 1**).

|  | | | | **Age-Specific Adjusted Odds Ratios** | | | |
| --- | --- | --- | --- | --- | --- | --- | --- |
| **Outcome** | **Risk Factor** | **Value** | **Adjusted Odds Ratio (vs. Non-Hispanic White)** | **At Age 20** | **At Age 40** | **At Age 60** | **At Age 80** |
| Testing in Emergency Department | Race/Ethnicity | Non-Hispanic Black |  | 2.30 [1.51—3.49] | 1.84 [1.52—2.23] | 1.54 [1.25—1.88] | 1.34 [0.95—1.87] |
|  |  | Hispanic |  | 1.90 [0.80—4.51] | 1.77 [1.07—2.95] | 1.44 [0.81—2.55] | 1.01 [0.41—2.51] |
|  |  | Other |  | 0.79 [0.46—1.36] | 0.64 [0.49—0.85] | 0.63 [0.45—0.86] | 0.73 [0.44—1.22] |
|  | Nbd. Income | $25,000 |  | 3.65 [1.90—7.03] | 3.35 [2.13—5.25] | 3.01 [1.89—4.79] | 2.64 [1.45—4.81] |
|  |  | $50,000 |  | 1.98 [1.18—3.32] | 2.04 [1.40—2.97] | 2.00 [1.36—2.94] | 1.87 [1.21—2.89] |
|  |  | $80,000 |  | 1.36 [0.98—1.87] | 1.41 [1.12—1.78] | 1.41 [1.11—1.79] | 1.37 [1.04—1.80] |
|  | Neighborhood Vehicle Access (% households) |  | 0.84 [0.46— 1.53] |  |  |  |  |
|  | Duration of Symptoms | 0-2 Days | 1.29 [0.95— 1.75] |  |  |  |  |
|  |  | 3-7 Days | 0.89 [0.66— 1.21] |  |  |  |  |
|  |  | 1-2 Weeks | 0.71 [0.52— 0.98] |  |  |  |  |
|  |  | >2 Weeks | 0.66 [0.47— 0.91] |  |  |  |  |
|  | Symptom Class | Class 2 | 0.39 [0.35— 0.45] |  |  |  |  |
|  |  | Class 3 | 0.78 [0.64— 0.95] |  |  |  |  |
|  |  | Class 4 | 0.51 [0.45— 0.58] |  |  |  |  |
|  |  | Class 5 | 0.55 [0.48— 0.63] |  |  |  |  |
|  |  | Class 6 | 0.87 [0.71— 1.05] |  |  |  |  |
| Test Positivity | Race/Ethnicity | Non-Hispanic Black |  | 1.81 [0.91—3.59] | 1.86 [1.40—2.48] | 2.04 [1.52—2.73] | 2.37 [1.54—3.65] |
|  |  | Hispanic |  | 0.26 [0.03—2.06] | 0.70 [0.26—1.86] | 1.23 [0.46—3.31] | 1.42 [0.35—5.69] |
|  |  | Other |  | 1.16 [0.50—2.74] | 0.65 [0.40—1.05] | 0.76 [0.44—1.29] | 1.84 [0.94—3.59] |
|  | Nbd. Income | $25,000 |  | 0.67 [0.20—2.22] | 0.94 [0.42—2.14] | 1.01 [0.44—2.31] | 0.83 [0.32—2.16] |
|  |  | $50,000 |  | 0.89 [0.36—2.20] | 1.20 [0.59—2.46] | 1.33 [0.64—2.73] | 1.19 [0.55—2.58] |
|  |  | $80,000 |  | 0.98 [0.56—1.70] | 1.17 [0.75—1.81] | 1.24 [0.80—1.94] | 1.19 [0.73—1.92] |
|  | Duration of Symptoms | 0-2 Days | 2.61 [1.14— 5.98] |  |  |  |  |
|  |  | 3-7 Days | 3.60 [1.57— 8.24] |  |  |  |  |
|  |  | 1-2 Weeks | 4.26 [1.85— 9.84] |  |  |  |  |
|  |  | >2 Weeks | 3.78 [1.63— 8.78] |  |  |  |  |
|  | Symptom Class | Class 2 | 1.67 [1.24— 2.25] |  |  |  |  |
|  |  | Class 3 | 3.11 [2.16— 4.47] |  |  |  |  |
|  |  | Class 4 | 4.12 [3.14— 5.41] |  |  |  |  |
|  |  | Class 5 | 6.52 [5.00— 8.50] |  |  |  |  |
|  |  | Class 6 | 10.00 [7.37—13.56] |  |  |  |  |
|  | Testing in Emergency Department |  | 0.78 [0.68— 0.90] |  |  |  |  |
| Hospitalization (Positive Cases) | Race/Ethnicity | Non-Hispanic Black |  | ** | ** | ** | ** |
|  |  | Hispanic |  | ** | ** | ** | ** |
|  |  | Other |  | ** | ** | ** | ** |
|  | Nbd. Income | $25,000 |  | ** | ** | ** | ** |
|  |  | $50,000 |  | ** | ** | ** | ** |
|  |  | $80,000 |  | ** | ** | ** | ** |
|  | Neighborhood Vehicle Access (% households) |  | 3.24 [0.22—47.02] |  |  |  |  |
|  | Duration of Symptoms | 0-2 Days | 0.73 [0.11— 4.66] |  |  |  |  |
|  |  | 3-7 Days | 0.66 [0.10— 4.26] |  |  |  |  |
|  |  | 1-2 Weeks | 0.66 [0.10— 4.30] |  |  |  |  |
|  |  | >2 Weeks | 0.46 [0.07— 3.15] |  |  |  |  |
|  | Symptom Class | Class 2 | 0.47 [0.22— 1.03] |  |  |  |  |
|  |  | Class 3 | 0.97 [0.39— 2.41] |  |  |  |  |
|  |  | Class 4 | 0.34 [0.17— 0.67] |  |  |  |  |
|  |  | Class 5 | 0.68 [0.35— 1.30] |  |  |  |  |
|  |  | Class 6 | 1.14 [0.56— 2.32] |  |  |  |  |
|  | Testing in Emergency Department |  | 5.38 [3.87— 7.48] |  |  |  |  |
|  | Current or Former Smoker |  | 0.96 [0.69— 1.34] |  |  |  |  |
|  | Cardiovascular Disease |  | 2.49 [1.66— 3.75] |  |  |  |  |
|  | Autoimmune Disease |  | 1.26 [0.87— 1.83] |  |  |  |  |
|  | Angiotensin Converting Enzyme Inhibitors |  | 0.74 [0.46— 1.21] |  |  |  |  |
|  | Angiotensin Receptor Blockers |  | 0.68 [0.40— 1.15] |  |  |  |  |
| ** Odds ratio estimates not reported due to limited sample size. | | | | | | | |

# **Supplemental Table 4**

Predicted probability of testing in the Emergency Department (ED), SARS-CoV-2 test positivity and hospitalization (among positive cases), stratified by race/ethnicity or neighborhood median income and age.

| **Testing in the Emergency Department** | | | **Test Positivity** | | | **Hospitalization (Positive Cases)** | | |
| --- | --- | --- | --- | --- | --- | --- | --- | --- |
| **Race/Ethnicity or Median Nbd. Income** | **Age** | **Probability [95% CI]** | **Race/Ethnicity or Median Nbd. Income** | **Age** | **Probability [95% CI]** | **Race/Ethnicity or Median Nbd. Income** | **Age** | **Probability [95% CI]** |
| Race/Ethnicity: Hispanic | 20 | 0.51 [0.339, 0.678] | Race/Ethnicity: Hispanic | 20 | 0.0163 [0.00288, 0.0874] | Race/Ethnicity: Hispanic | 20 | 0.00118 [<0.001, 0.865] |
|  | 40 | 0.492 [0.388, 0.596] |  | 40 | 0.0644 [0.0294, 0.136] |  | 40 | 0.0857 [0.00413, 0.679] |
|  | 60 | 0.524 [0.406, 0.638] |  | 60 | 0.116 [0.0541, 0.232] |  | 60 | 0.52 [0.135, 0.883] |
|  | 80 | 0.604 [0.421, 0.762] |  | 80 | 0.104 [0.0349, 0.27] |  | 80 | 0.646 [0.121, 0.96] |
| Race/Ethnicity: NH Black | 20 | 0.527 [0.449, 0.604] | Race/Ethnicity: NH Black | 20 | 0.11 [0.0708, 0.167] | Race/Ethnicity: NH Black | 20 | 0.0589 [0.013, 0.229] |
|  | 40 | 0.5 [0.464, 0.536] |  | 40 | 0.146 [0.122, 0.173] |  | 40 | 0.176 [0.112, 0.265] |
|  | 60 | 0.554 [0.517, 0.591] |  | 60 | 0.182 [0.154, 0.215] |  | 60 | 0.454 [0.352, 0.56] |
|  | 80 | 0.683 [0.625, 0.736] |  | 80 | 0.216 [0.17, 0.271] |  | 80 | 0.788 [0.628, 0.891] |
| Race/Ethnicity: NH White | 20 | 0.329 [0.293, 0.366] | Race/Ethnicity: NH White | 20 | 0.0738 [0.0565, 0.0959] | Race/Ethnicity: NH White | 20 | 0.0303 [0.0114, 0.078] |
|  | 40 | 0.346 [0.329, 0.363] |  | 40 | 0.0948 [0.0849, 0.106] |  | 40 | 0.15 [0.109, 0.202] |
|  | 60 | 0.438 [0.419, 0.457] |  | 60 | 0.11 [0.0978, 0.122] |  | 60 | 0.368 [0.309, 0.43] |
|  | 80 | 0.611 [0.582, 0.639] |  | 80 | 0.115 [0.0975, 0.134] |  | 80 | 0.53 [0.442, 0.616] |
| Race/Ethnicity: Other | 20 | 0.277 [0.202, 0.366] | Race/Ethnicity: Other | 20 | 0.0903 [0.0485, 0.162] | Race/Ethnicity: Other | 20 | 0.00269 [<0.0001, 0.21] |
|  | 40 | 0.252 [0.214, 0.295] |  | 40 | 0.0709 [0.0504, 0.0988] |  | 40 | 0.0682 [0.0143, 0.27] |
|  | 60 | 0.322 [0.271, 0.379] |  | 60 | 0.0934 [0.0638, 0.135] |  | 60 | 0.307 [0.139, 0.549] |
|  | 80 | 0.518 [0.417, 0.617] |  | 80 | 0.197 [0.129, 0.288] |  | 80 | 0.375 [0.172, 0.633] |
| Nbd. Income: $ 25,000 | 20 | 0.605 [0.507, 0.695] | Nbd. Income: $ 25,000 | 20 | 0.0353 [0.0164, 0.0745] | Nbd. Income: $ 25,000 | 20 | 0.064 [0.00456, 0.505] |
|  | 40 | 0.599 [0.54, 0.655] |  | 40 | 0.0421 [0.0264, 0.0667] |  | 40 | 0.232 [0.0821, 0.505] |
|  | 60 | 0.664 [0.607, 0.717] |  | 60 | 0.0468 [0.0291, 0.0746] |  | 60 | 0.536 [0.287, 0.769] |
|  | 80 | 0.781 [0.71, 0.84] |  | 80 | 0.0485 [0.0267, 0.0866] |  | 80 | 0.794 [0.402, 0.957] |
| Nbd. Income: $ 50,000 | 20 | 0.396 [0.35, 0.444] | Nbd. Income: $ 50,000 | 20 | 0.0596 [0.0425, 0.0828] | Nbd. Income: $ 50,000 | 20 | 0.058 [0.018, 0.172] |
|  | 40 | 0.415 [0.394, 0.436] |  | 40 | 0.0778 [0.0675, 0.0895] |  | 40 | 0.192 [0.134, 0.269] |
|  | 60 | 0.507 [0.484, 0.53] |  | 60 | 0.0904 [0.078, 0.105] |  | 60 | 0.401 [0.326, 0.481] |
|  | 80 | 0.668 [0.633, 0.701] |  | 80 | 0.094 [0.076, 0.116] |  | 80 | 0.578 [0.441, 0.704] |
| Nbd. Income: $ 80,000 | 20 | 0.305 [0.272, 0.341] | Nbd. Income: $ 80,000 | 20 | 0.0805 [0.0628, 0.103] | Nbd. Income: $ 80,000 | 20 | 0.0201 [0.0076, 0.052] |
|  | 40 | 0.32 [0.304, 0.335] |  | 40 | 0.102 [0.0915, 0.112] |  | 40 | 0.129 [0.0943, 0.175] |
|  | 60 | 0.41 [0.391, 0.428] |  | 60 | 0.116 [0.105, 0.129] |  | 60 | 0.359 [0.304, 0.419] |
|  | 80 | 0.586 [0.559, 0.613] |  | 80 | 0.122 [0.106, 0.141] |  | 80 | 0.524 [0.445, 0.601] |
| Nbd. Income: $120,000 | 20 | 0.258 [0.213, 0.31] | Nbd. Income: $120,000 | 20 | 0.101 [0.0728, 0.139] | Nbd. Income: $120,000 | 20 | 0.00406 [<0.0001, 0.026] |
|  | 40 | 0.26 [0.239, 0.281] |  | 40 | 0.117 [0.102, 0.133] |  | 40 | 0.075 [0.0402, 0.136] |
|  | 60 | 0.338 [0.313, 0.363] |  | 60 | 0.129 [0.112, 0.148] |  | 60 | 0.353 [0.281, 0.432] |
|  | 80 | 0.517 [0.48, 0.554] |  | 80 | 0.137 [0.115, 0.163] |  | 80 | 0.554 [0.454, 0.649] |
